# Lycopene supplementation: effects on oxidative stress, sex hormones, gonads and thyroid tissue in tilapia *Oreochromis niloticus* during Harness^®^ exposure

**DOI:** 10.3389/fphys.2023.1237159

**Published:** 2023-08-11

**Authors:** Rania F. Ismail, Mohamed Hamed, Alaa El-Din H. Sayed

**Affiliations:** ^1^ National Institute of Oceanography and Fisheries, NIOF, Cairo, Egypt; ^2^ Department of Zoology, Faculty of Science, Al-Azhar University (Assiut branch), Assiut, Egypt; ^3^ Zoology Department, Faculty of Science, Assiut University, Assiut, Egypt; ^4^ Molecular Biology Research and Studies Institute, Assiut University, Assiut, Egypt

**Keywords:** acetochlor, thyroid follicles, gonads, fish, antioxidants, T, E2

## Abstract

Harness^®^ is a commercial herbicide that contains acetochlor at a concentration of 84% as an active ingredient. Ubiquitous, persistent, and substantial uses of Harness^®^ in agricultural processes have resulted in the pollution of nearby water sources, posing a threat to various aquatic biotas, including fish. The effects of Harness^®^ toxicity on fish health are little known. So, this study aimed to describe the impact of herbicide Harness^®^ on the oxidative stress and reproductive and thyroid performance of male and female tilapia (*Oreochromis niloticus*) and also investigate the prospective role of the natural antioxidant lycopene supplementation in dismissing the adverse properties of Harness^®^. Antioxidant enzyme (catalase, superoxide dismutase, and total antioxidant capacity) and hormone measurements (T, E2, T3, and T4) were carried out, and gonadal and thyroid follicle histological sections were examined as a method to investigate the effects of Harness^®^ toxicity on fish. Male and female tilapia were exposed to 10 μmol/L and 100 μmol/L of Harness^®^ and treated with 10 mg lycopene/kg for 15 days of exposure. Our results demonstrated that the antioxidant enzyme activity was altered by Harness exposure and serum T for both males and females dropped; also, female E2 levels decreased, but male E2 increased. Exposure to higher dose of Harness^®^ induced elevation in both T3 and T4 levels, although the low exposure dose stimulated T4 levels. Harness^®^ exposure prompted histological variations and degenerative changes in testicular, ovarian, and thyroid follicle tissues. Lycopene supplement administration diminished oxidative stress induced by Harness^®^, alleviating its endocrine disparaging effects by neutralizing T3, T4, T, and E2 and ameliorating the histological structure of gonadal and thyroid tissues. In conclusion, lycopene supplementation was preformed to normalize the alterations and oxidative damage caused by Harness^®^ in Nile tilapia, suggesting that lycopene-supplemented diet functioned as potent antioxidants and had the ability to alleviate oxidative stress and thyroid and reproductive toxicity caused by herbicide Harness^®^. Moreover, it is crucial to take appropriate care when consuming herbicides to defend the aquatic environment.

## 1 Introduction

Water contamination has been one of the world’s most serious topics in latest decades, which arises mainly from urbanization, in addition to agricultural, industrial, domestic, and human activities (Arif et al., 2020; Haredi et al., 2020). Contaminated water decreases the reliability of water resources and is a threat to aquatic species (Fatima et al., 2020; Hamed et al., 2023). The extensive and global use of herbicides in agriculture has led to the pollution of adjacent water resources, posing a threat to several aquatic biota such as fish (Guo et al., 2008; Sayed et al., 2023a).

Harness^®^ is a commercial herbicide that mainly comprises acetochlor (ACT) with 84% of its concentration as an active ingredient (Heydens et al., 2010). Harness^®^ is an herbicide frequently used in fighting annual weeds and grass and is used for various crops worldwide (Hoogeweg et al., 2020). Consistent with the United States Environmental Protection Agency (USEPA), Harness^®^ is the third most marketable herbicide extensively used in global agriculture (Chang et al., 2020).

Increasing concern over the impact of pesticides and man-made chemicals on the typical function of endocrine systems has been reported for many aquatic organisms. Several chemicals that have been acknowledged as endocrine disruptor chemicals (EDCs) are pesticides (Brown et al., 2004; Weis, 2014; Mohamed et al., 2022). In fish, gonadal development, successful gametogenesis, and spawning are regulated through the endocrine axis. When the hypothalamic–pituitary–gonadal axis is disrupted, there is possibility of adverse effects on gonadal progress, egg and sperm creation, intersex, or even sterility (Kidd et al., 2007). Therefore, persistent existence of sex steroid endocrine disruptors in water bodies can lead to population drops and, under severe circumstances, excision and extirpation of species (Kidd et al., 2007). In addition to the sex steroids, environmental contaminants can influence the thyroid axis. In fish, thyroid hormones are accepted as significant regulators in growth, differentiation, metabolism, and adaptation to salinity (Orozco et al., 2002).

It was reported that ACT is a toxic constituent for fish, with LC50 0.5 mgL^−1^ for rainbow trout (*Oncorhynchus mykiss*) and 1 mgL^−1^ for bluegill (Helfrich et al., 2009). ACT is extremely harmful to fish as it can initiate reproductive toxicity, immune toxicity, and endocrine toxicity in zebrafish (Jiang et al., 2016). Moreover, ACT generates DNA injury and induces oxidative stress in bighead carp (*Aristichthys nobilis*) (Mahmood et al., 2022). It was reported that ACT is a thyroid chemical disorder-causing mediator (Yang et al., 2022). It can modify the expression of the hypothalamic–pituitary–thyroid (HPT) axis-related genes (Li et al., 2009; Yang et al., 2016; Guo et al., 2020) and change the whole body thyroid hormone content in zebrafish larvae (Guo et al., 2020). ACT is thought to be a carcinogen and endocrine-disrupting agent (Zhang et al., 2019). The low concentrations of ACT displayed an estrogenic effect in zebrafish (Zhang et al., 2020); when zebrafish were exposed to higher doses of ACT, the ovarian resistance to oxidative stress reduced and the ovarian growth stopped (Zhang et al., 2020).

Lycopene (Lyc) is a natural carotenoid with red color pigment and existing in a variety of fruits and vegetables (Kelkel et al., 2011; Tanaka et al., 2012). Lycopene has various positive effects on human health because of its antioxidant activity; it is involved in the cure of liver damage, metabolic disorders, male infertility, and cancer (Grabowska et al., 2019). In addition, Lyc can boost the transcription factors Nrf2 and NF-jB that have considerable function in the triggering of phase II detoxifying enzymes, which are associated with the antioxidant defense mechanisms (Linnewiel et al., 2009; Palozza et al., 2012; Sayed et al., 2021).

In fish, lycopene has recently attracted interest with its possible modes of action in weakening oxidative stress prompted by aquatic environmental contaminants because of its extremely effective antioxidant scavenging role (Sahin et al., 2014; Dawood et al., 2020; Hamed et al., 2020), and lycopene boosted fish immune response and antioxidant capacity (Abd El-Gawad et al., 2019; Hamed et al., 2022; Kesbiç et al., 2022).

Lycopene could defend the cells from the DNA injury caused by oxidative stress owing to its capability to reduce reactive oxygen species (ROS) and avoid mutation, which is the chief source of chronic diseases (Goralczyk and Siler, 2003). In addition, lycopene showed efficiency in the amelioration of thyroid gland organization along with DNA damage, through its antioxidant properties (Abdul-Hamid and Salah, 2013). Dawood et al. (2020) indorsed lycopene supplementation to recover the oxidative stress and relieve the hemato-immunological modifications that appeared in fish exposed to pollutants of the aquatic environment (heavy metals and insecticides).

Former studies have revealed that Harness^®^ (active ingredient: acetochlor) has an extensive range of effects, such as hemato-biochemical parameters, genotoxicity, and histopathological changes (Sayed A. E.-D. H. et al., 2022). Consequently, it is crucial to comprehend the potential impacts of Harness^®^ on the health and productivity of important economic fish species such as tilapia and also to evaluate the potential detoxifying effect of lycopene. Consequently, the existent study aimed to define the impact of Harness^®^ on oxidative stress, thyroid toxicity, and reproductive dysfunction. Antioxidant enzymes, thyroid and sex steroid hormone production with the detection of thyroid follicles, and testicular and ovarian tissue histological alterations are used as bioindicators. Another purpose is to investigate the effect of dietary lycopene supplementation to relieve the oxidative stress and the stabilization of the alterations induced by acetochlor exposure.

## 2 Materials and methods

### 2.1 Chemicals

The herbicide Harness^®^ used in this study was purchased from Monsanto Company that contains 84% acetochlor (C14H20NO2Cl; MW. 269.77) as an active component. The lycopene red pigment (natural carotenoid) was purchased from Sigma-Aldrich (Cairo, Egypt).

### 2.2 Fish

Healthy Nile tilapia, *O. niloticus*, with an average total weight of 2.664 ± 0.57 g and 4.17 ± 0.72 cm total length, were brought from the tilapia farm and moved to the acclimation tanks in the Fish Biology and Pollution Laboratory, Zoology Department, Faculty of Science, Assiut University. They were healthy and free from parasites according to AFS-FHS (2004). During acclimation, fish were retained in 60 L rectangular tanks with fresh aerated water and were fed a 5% of body weight commercial diet daily (SKRETTING, Egypt). The water was completely replaced, and feces and leftover food were removed daily, and the photoperiod 12:12 light–darkness regime was followed for 2 weeks.

### 2.3 Experimental design

Fish were allocated into five experimental groups in triplicate tanks (10 fish/tank) as follows.

The first group identified as a control group was fed a commercial diet without any added lycopene or Harness^®^ exposure. In the second group (H1), fish were fed a commercial diet and subjected to 10 μmol/L Harness^®^. In the third group (H1Lyc), fish were fed a commercial diet accompanied with 10 mg LYC/kg fish weight and subjected to 10 μmol/L Harness^®^. In the fourth group (H2), fish were fed a commercial diet and subjected to 100 μmol/L Harness^®^. In the fifth group (H2Lyc), fish were fed a supplemented commercial diet with 10 mg lycopene/kg fish weight and treated with 100 μmol/L Harness^®^.

During the experimental period, 100% of the rearing water was substituted daily with each required herbicide concentration consistent with the experimental strategy. Harness^®^ dosages were selected according to the work of Xu et al. (2016) and Sayed A. E.-D. H. et al. (2022), while lycopene amounts were selected according to the work of Yonar (2012).

After 15 days of experimental exposure, six fish (per replicate from every group) were arbitrarily chosen and benumbed with ice (Hamed et al., 2019) to reduce the stress of further handing out. Blood was withdrawn from the caudal vein and then allowed to clot at 4°C. The blood samples, then, were centrifuged at 4,000 g for 10 min to obtain serum. Fish were dissected, and then, the whole head and gonads were placed in a 4% buffered formalin solution until histology was conducted.

### 2.4 Antioxidant enzyme activities

Superoxide dismutase (SOD) was measured based on its capability to prevent the phenazine methosulphate-mediated reduction of nitroblue tetrazolium dye to form a red product consistent with the work of Nishikimi et al. (1972). Catalase (CAT) was determined based on the fact that 3,5-dichloro-2-hydroxybenzene sulfonic acid could rapidly dismiss the degradation reaction of hydrogen peroxide catalyzed by CAT and react with the residual hydrogen peroxide to generate a yellow product (Aebi, 1984). Total antioxidant capacity (TAC) was measured as stated by Koracevic et al. (2001).

### 2.5 Hormone measurements

Estradiol (E2) was assessed using the ELISA kit (CAN-E−430, Diagnostics Biochem Canada Inc., Ontario, Canada). Testosterone (T) was also evaluated using a test kit (CAN-TE-250) as described by Check et al. (1995). For thyroid hormones, T3 was assessed using the T3 ELISA kit (EIAab, no. E0453f, United States), and for T4 evaluation, the T4 ELISA kit was used (MBS701162, BioSource, United States), in accordance with the manufacturer’s guidelines. All hormones were assessed at 450 nm using an automatic immunodiagnostic analyzer (Sorin Biomedica, Model: 0-2730; S/N = 0654, Chemila SP.A., Italy) (Sayed E.-D. H. et al., 2022).

### 2.6 Histological analysis

For thyroid follicle examination, the fixed fish heads were placed in 10% EDTA (PH 7.4) solution for 1 week for decalcification. Then, fixed gonad and EDTA-treated head samples were dehydrated through arising grades of ethanol and then were cleared and embedded in paraffin wax. For thyroid follicle sections, serial frontal sections were cut at 7 μm from the ventral side of the head. The embedded gonad samples were sectioned at 5 µm thickness; the samples were stained with H&E stain and then observed microscopically (Hamed et al., 2021; Sayed E.-D. H. et al., 2022; Sayed et al., 2023b). For scoring each histopathological parameter, six sections of four fish from each treatment were randomly selected and labeled as follows: control, unchanged (0–2); mild, + (>2–10%) area of section; moderate, ++ (>10–40%) partition area; and severe, +++ (>40% partition area) (Hamed et al., 2021). The sections were examined under an Olympus microscope (model BX50F4, Olympus Optical Co., Ltd., Tokyo, Japan).

### 2.7 Statistical analysis

Data are stated as mean ± SE. Significant changes amongst the treatments and control groups were verified using one-way analysis of variance (ANOVA) and, subsequently, the Tukey-HSD test for multiple comparisons. The analysis was achieved using the SPSS^®^ version 23.0 package (SPSS, Richmond, VA, USA) as described by Dytham (2011).

## 3 Results

### 3.1 Antioxidant enzyme activities

**FIGURE 1:**
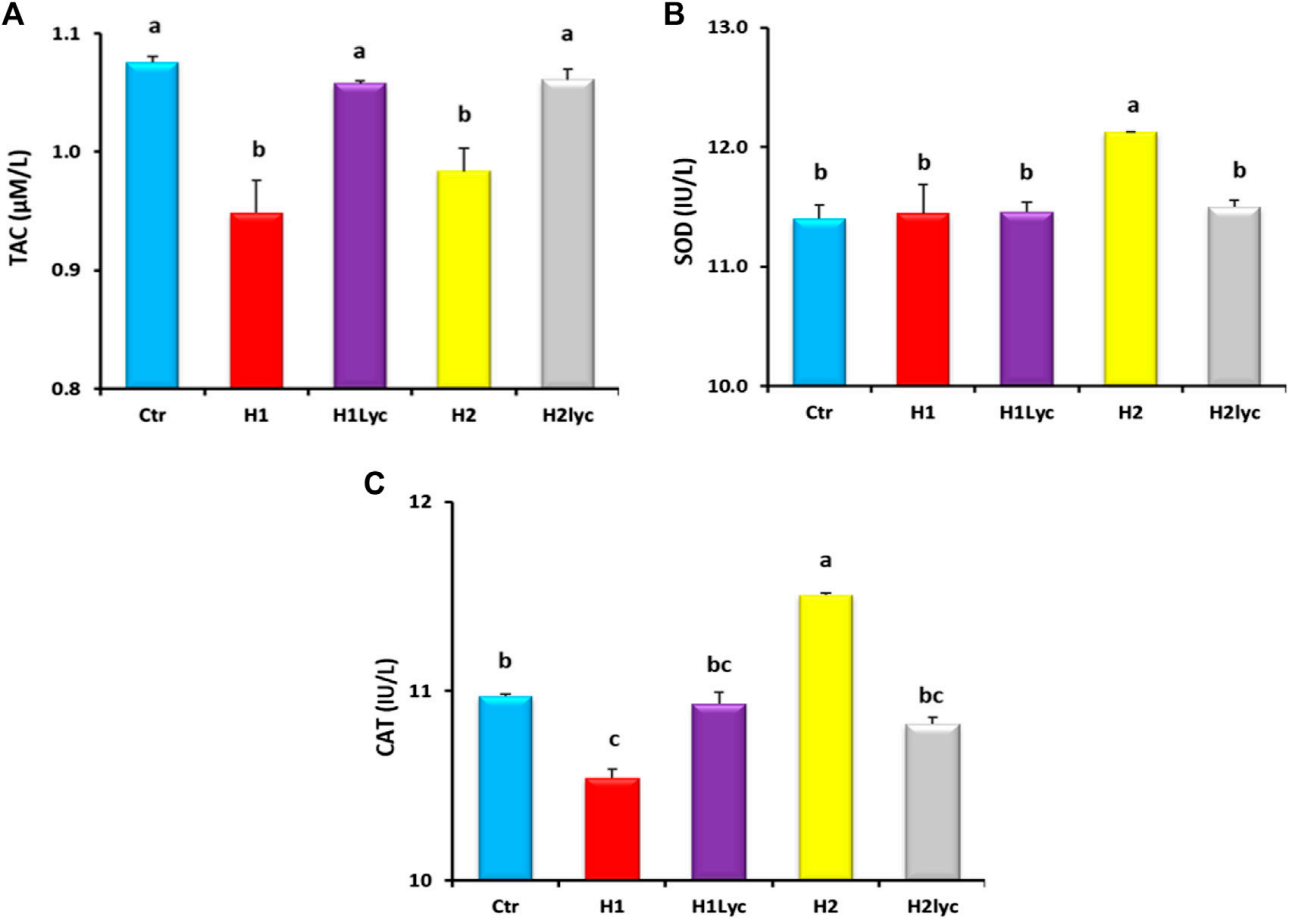
Antioxidant enzyme activity in Nile tilapia after 15 days of exposure: **(A)** TAC enzyme, **(B)** SOD enzyme and **(C)** CAT enzyme. Ctr, H1, H1Lyc, H2 and H2Lyc. Results are presented as mean ± SE. Values with diverse superscript letters are significantly changed (*p* < 0.05).

Serum TAC levels significantly decreased (*p < 0.05*) after exposure to Harness^®^ for groups H1 and H2 compared to the control (ctr), while TAC levels for the groups supplemented with lycopene (H1Lyco and H2Lyco) were insignificant (*p < 0.05*) with the control group as displayed in Figure 1A.

A significant increase (*p < 0.05*) in serum SOD was detected in high concentrations of Harness^®^ (group H2) compared to the other experimental groups (Figure 1B). Lycopene co-treatment suppressed the Harness^®^-induced increase, resulting in a significantly less SOD activity than for the Harness^®^ alone, and restored SOD levels to the control levels (Figure 1B). For the fish group exposed to 10 μmol/L Harness^®^ alone or 10 μmol/L Harness^®^ and lycopene supplement, no significant changes in SOD were detected in comparison to the control.

Exposure to 10 μmol/L Harness^®^ alone induced a statistically significant (*p < 0.05*) decrease in blood CAT activity compared to the control, while high-dose exposure of 100 μmol/L Harness^®^ induced a significant increase in CAT activity. The Harness®-exposed groups treated with lycopene displayed a marked normalization of serum CAT activity with no statistically significant effect compared to the control group level (Figure 1C).

### 3.2 Thyroid hormonal activity

**FIGURE 2:**
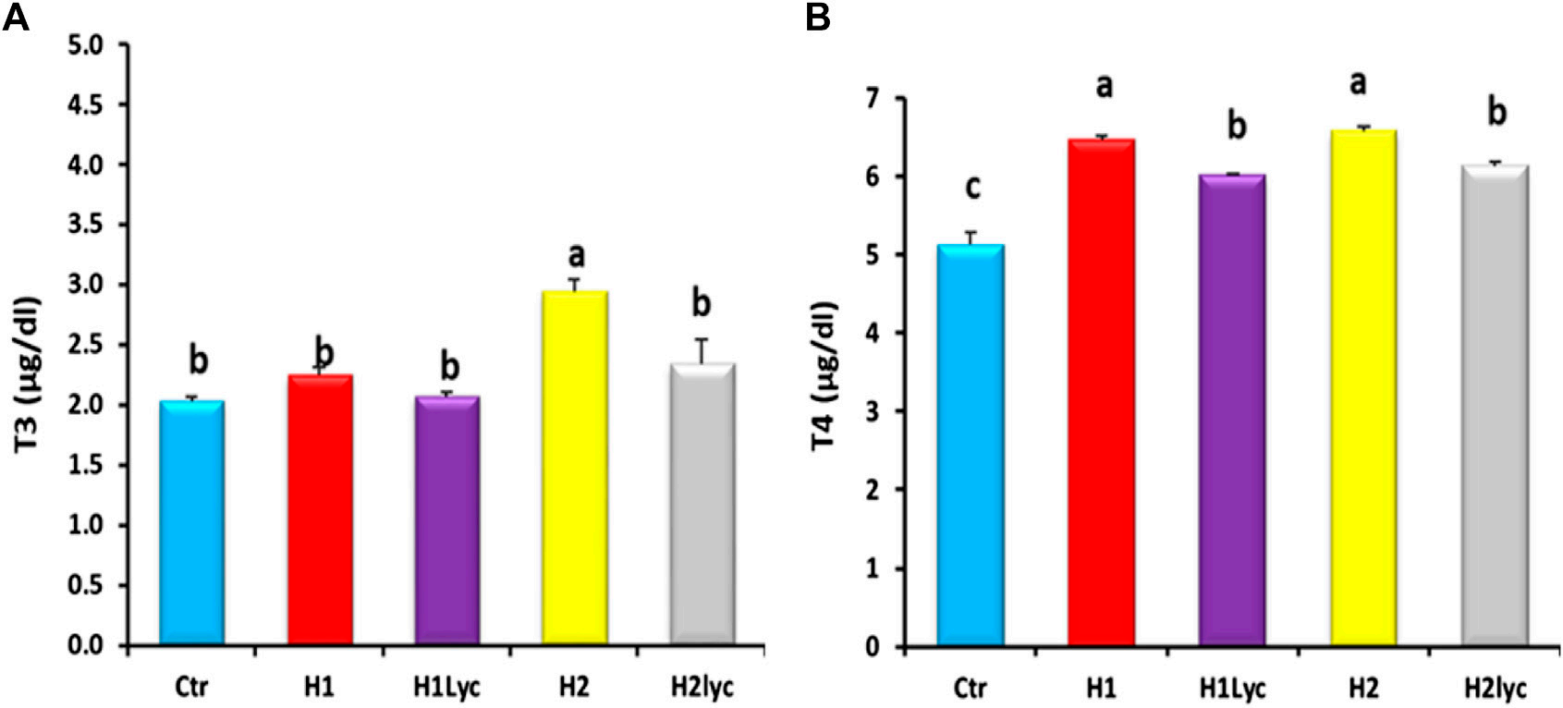
Thyroid hormonal activity (T3 and T4) in Nile tilapia after 15 days of exposure, Ctr, H1, H1Lyc, H2, and H2Lyc. Data are accessible as mean ± SE. Means with unalike letters are significantly different (*p* < 0.05).

Thyroid hormones (T3 and T4) were detected in serum of both males and females and displayed comparable values without significant differences between male and female samples. Serum T3 levels of the H2 group were significantly (*p < 0.05*) higher than those of the control, H1, H1Lyc, and H2lyc, while the co-treatment with lycopene (group H2lyc) restored T3 to the control level (Figure 2A). For serum T4, the two exposure doses of Harness induced significant elevation (*p <* 0.05) in serum T4 compared to the control, while for Harness co-treated with lycopene groups (H1Lyc and H2lyc), a significant (*p <* 0.05) decrease in T4 levels was reported but not the same control levels (Figure 2A).

### 3.3 Sex steroids activity

**FIGURE 3:**
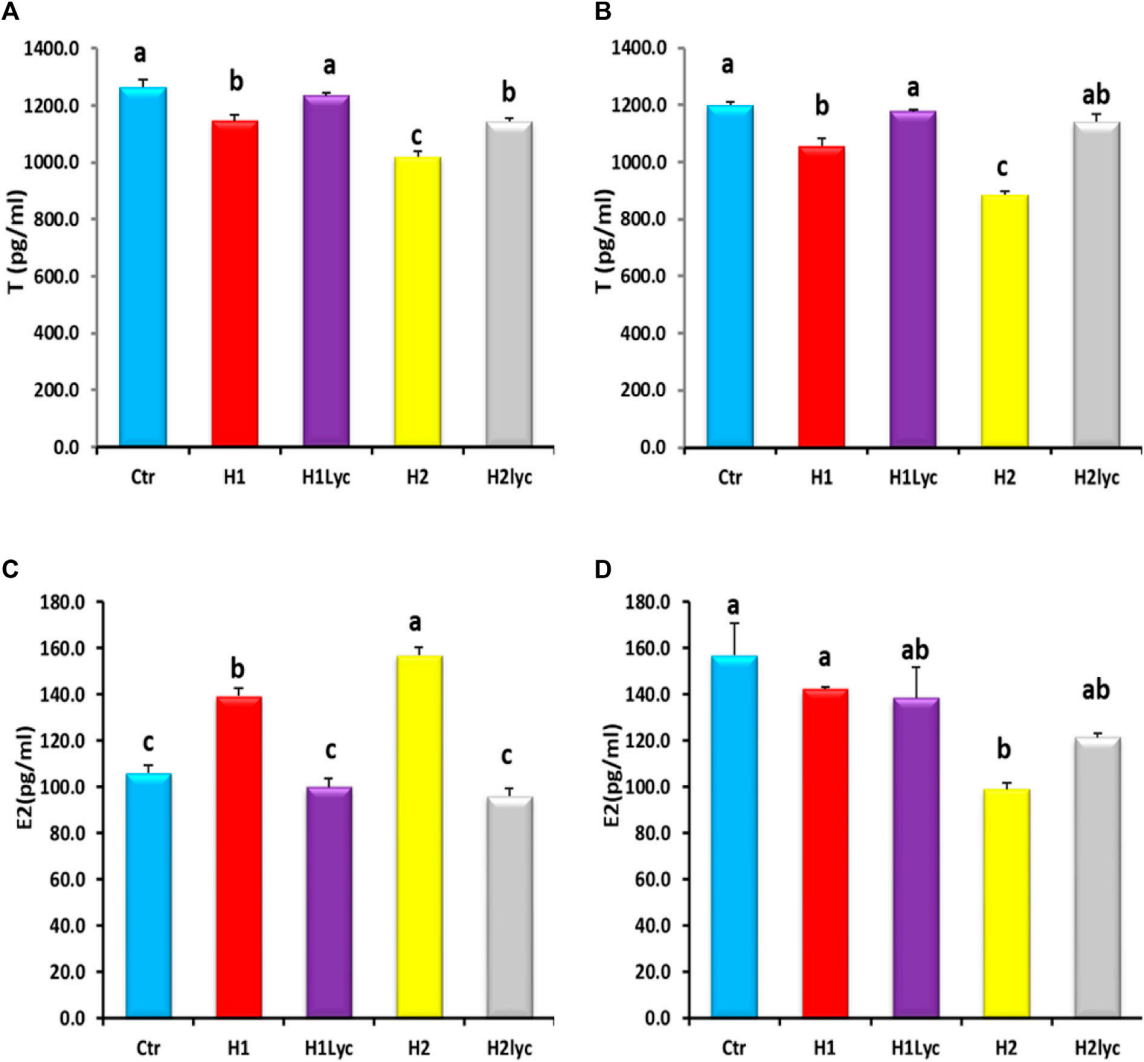
Testosterone (T) and estradiol (E2) activities in Nile tilapia after 15 days of exposure: **(A)** male serum T, **(B)** female serum T, **(C)** male serum E2, and **(D)** female serum E2. Ctr, H1, H1Lyc, H2, and H2Lyc. Data are accessible as mean ± SE. Values with unalike letters are significantly different (*p <* 0.05).

The serum testosterone (T) for both male and females displayed significant (*p <* 0.05) reduction for both exposure doses when compared to the control. This reduction was herbicide concentration dependent (Figures 3A, B). In male fish, the co-treatment with lycopene was able to restore the T levels of male to the control level for the low dose of Harness^®^ (H1Lyc), while for the high dose of Harness^®^+lycopene (H2Lyc), the T serum level was significantly higher than H2 but less than the control level (Figure 3A), while for female fish, lycopene supplementation restored T levels as control (Figure 3B).

A significant (*p* < 0.05) increase in serum E2 levels was detected in male fish by the end of exposure time (Figure 3C), and this elevation was also Harness^®^ dose dependent. The highest increase in serum E2 levels was found in fish subjected to H2 dose. The lycopene supplementation neutralized the E2 levels to the control levels (Figure 3C).

For female serum E2, no significant changes were detected for the low exposure dose (H1), and E2 levels were comparable to the control, while the high exposure dose (H2 group) displayed a significant (*p* < 0.05) suppression of serum E2 compared to the control (Figure 3D).

### 3.4 Histological changes of thyroid follicles

**FIGURE 4:**
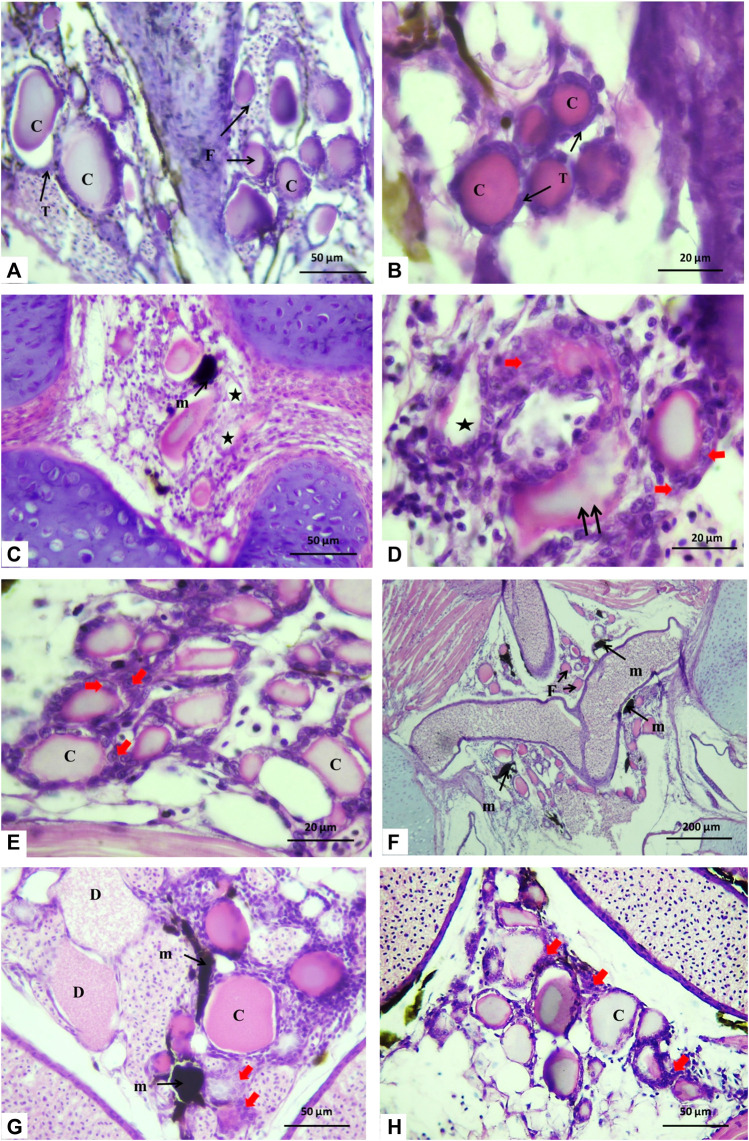
Histological sections of Nile tilapia taken through the glossopharyngeal region showing thyroid follicles after 15 days of exposure. **(A)** Control group displaying thyroid follicle (F), thyrocyte (T), and colloid (C). **(B)** Magnification of thyroid follicles in the control group displaying cuboidal epithelium thyrocytes (T) and a homogeneously eosin-stained colloid (C). **(C, D)** H1 group showing the melanomacrophage center (m) with colloid depletion (*), disorganized and irregular follicle shape (double arrows), and hypertrophic thyrocytes (red arrows). **(E)** In the H1Lyc group, the thyroid follicles displayed similar results as the control group with some hypertrophy in the thyrocytes (red arrows). **(F, G)** H2 fish group, with an increasing number of the melanomacrophage center (m), hypertrophy in the thyrocytes (red arrows), and increasing degeneration in follicle colloid (D). **(H)** Thyroid follicles of the H2Lyc fish group and thyroid tissue displaying some hypertrophic thyrocytes (red arrows), H&E stain.

The histological inspections displayed that the thyroid tissue, which was unencapsulated, consisted of scattered follicles of variable size diffusely distributed beneath the pharyngeal region, positioned at the dorsal and lateral aspects of the ventral aorta. The follicles were detected to be bound by connective tissue. Serial sections in the control group showed that the thyroid follicles had colloid-filled lumens and were enclosed by simple cuboidal cells (thyrocytes) with a rounded nucleus (Figures 4A, B).

For the fish group H1 (10 μmol Harness^®^/L), the sections showed asymmetrical-shaped follicles lined by a layer of follicular cells developing hyperplastic follicles. Colloid depletion of some follicles was observed, and some melanomacrophage centers (MMCs) were recognized with hypertrophy in thyrocytes (Figures 4C, D).

For the fish group co-treated with lycopene (H1lyc), the thyroid follicles displayed normal histological structure as the control group with some hypertrophy in the thyrocytes in few follicles (Figure 4E).

For the fish group H2 (100 μmol Harness^®^/L), a reduction in the number of thyroid follicles was recorded with an increasing number of MMCs, hyperplastic follicles with hypertrophy in the thyrocytes, and number of degenerated follicles in the colloid (Figures 4F, G). When the same Harness^®^ dose was co-treated with lycopene (H2lyc), the thyroid follicles exhibited a normal histological structure as in the control group, with some hypertrophy in the thyrocytes, and few thyroid follicles displayed vacuolated colloid while others were lacking colloid (Figure 4H). Semi-quantitative scoring for histological changes in the thyroid follicles displayed in Table 1.

**TABLE 1:** Semi quantitative scoring of the histopathology in the thyroid follicles, female gonads, and male gonads of *Oreochromis niloticus* exposed to Harness^®^.

Histopathological lesion	Control	Treated group			
		H1	H1Lyc	H2	H2Lyc
Thyroid follicles					
Colloid depletion of some follicles	**-**	**++**	**-**	**+++**	**+**
Increasing melanomacrophage centers	**-**	**++**	**-**	**+++**	**+**
Hypertrophic thyrocytes	**-**	**+**	**+**	**+++**	**+**
Hyperplastic follicles	**-**	**+**	**-**	**+++**	**_**
Gonad female					
Displayed few atretic follicles	**-**	**+**	**-**	**++**	**-**
Increasing number of oocytes	**-**	**+**	**-**	**++**	**-**
Increasing number of atretic follicles	**-**	**++**	**+**	**+++**	**+**
Less number of the secondary growth phase	**-**	**+**	**-**	**+++**	**+**
Gonad male					
Decrease in spermatozoa amount	**-**	**+**	**-**	**++**	**-**
Increasing melanomacrophage centers	**-**	**++**	**-**	**+++**	**+**
Necrosis	**-**	**+**	**-**	**++**	**-**

–, normal; +, mild (<10%); ++, moderate (10%–50%); +++, severe (>50%).

### 3.5 Histological changes of gonads

**FIGURE 5:**
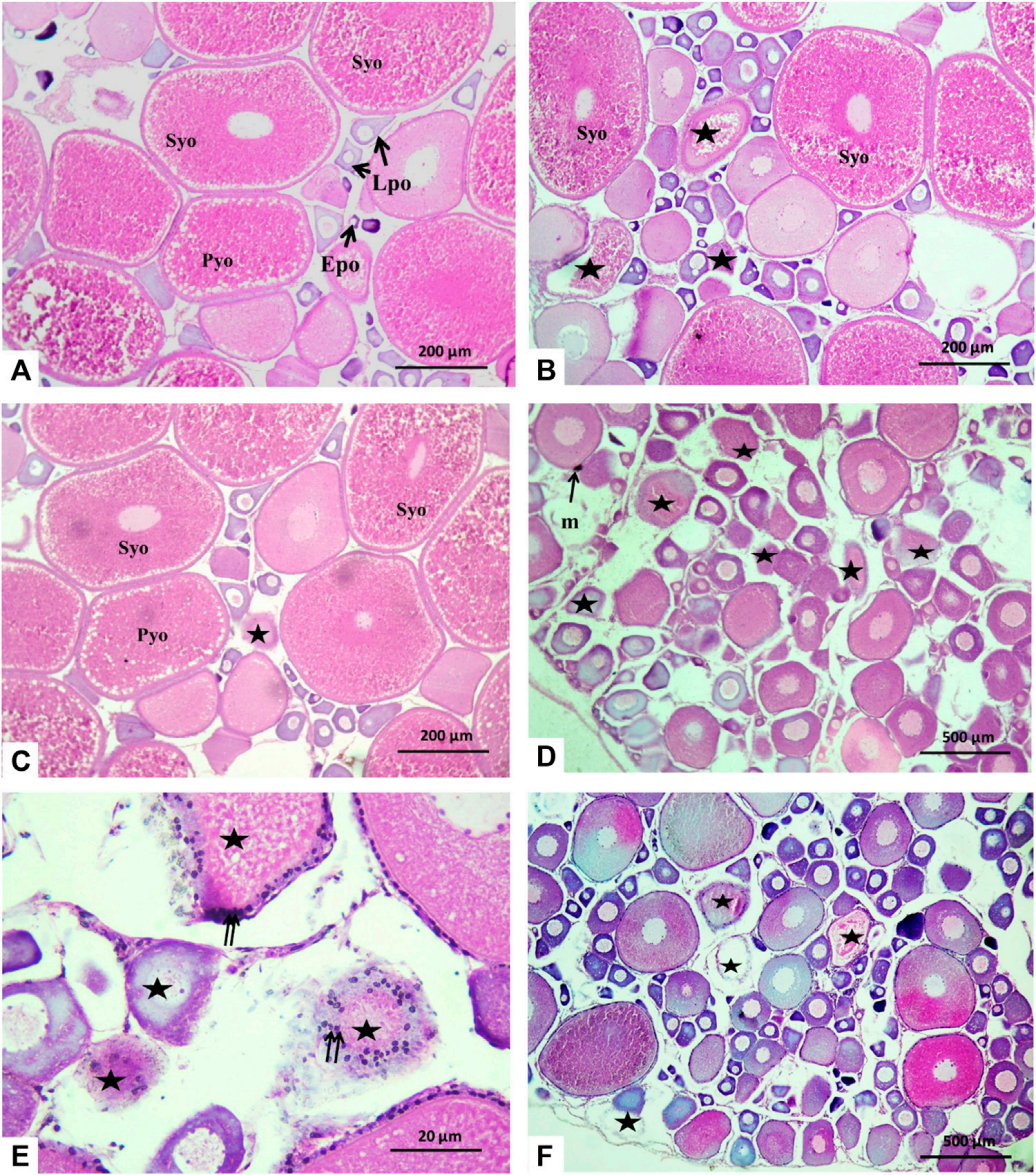
Histological cross section in Nile tilapia ovaries after 15 days of exposure. **(A)** Control group displaying different oocyte growth stages; early perinucleolar oocytes (EPOs), late perinucleolar oocytes (LPOs), primary yolk oocytes (PYOs), secondary yolk oocytes (SYOs), and tertiary yolk oocytes (TYOs). **(B)** H1 fish group showing the number of atretic oocytes (*). **(C)** In the H1Lyc fish group, the sections displayed few atretic oocytes (*). **(D, E)** H2 fish group with an increasing number of atretic oocytes (*), some melanomacrophage center (m), and irregular and damaged oocyte follicles with hypertrophic follicular cells (double arrow). **(F)** H2Lyc fish group with a smaller number of atretic oocytes (*) (H&E staining).

The histology microphotographs of the ovaries displayed the control group with different oocyte growth stages including the primary growth phase with early and late perinucleolar oocytes, in addition to the secondary growth phase including primary yolk oocytes, secondary yolk oocytes, and tertiary yolk oocytes (Figure 5A)**.** For the fish group exposed to 10 μm/L Harness^®^, ovaries displayed few atretic follicles and also an increasing number of oocytes of the primary growth phase with a smaller number of the secondary growth phase in comparison to the control (Figure 5B). In fish group H1Lyc, the ovarian histology microphotographs were similar to those of the control (Figure 5C). For the fish group exposed to 100 μmol/L Harness^®^, there was a significantly repressed effect on ovarian progress. The number of secondary growth phases and late stage decreased with an increasing number of perinucleolar oocytes compared to that of the control group; in addition, an increase in the number of atretic follicles was observed (Figures 5D, E). For lycopene-supplemented group H2Lyc, the ovaries showed less number of atretic oocytes compared to the H2 group, with a substantial number of perinucleolar oocytes compared to the control group (Figure 5F). The ovarian alterations of different treatment groups were assessed using Semi-quantitative scoring as described in Table 1.

**FIGURE 6:**
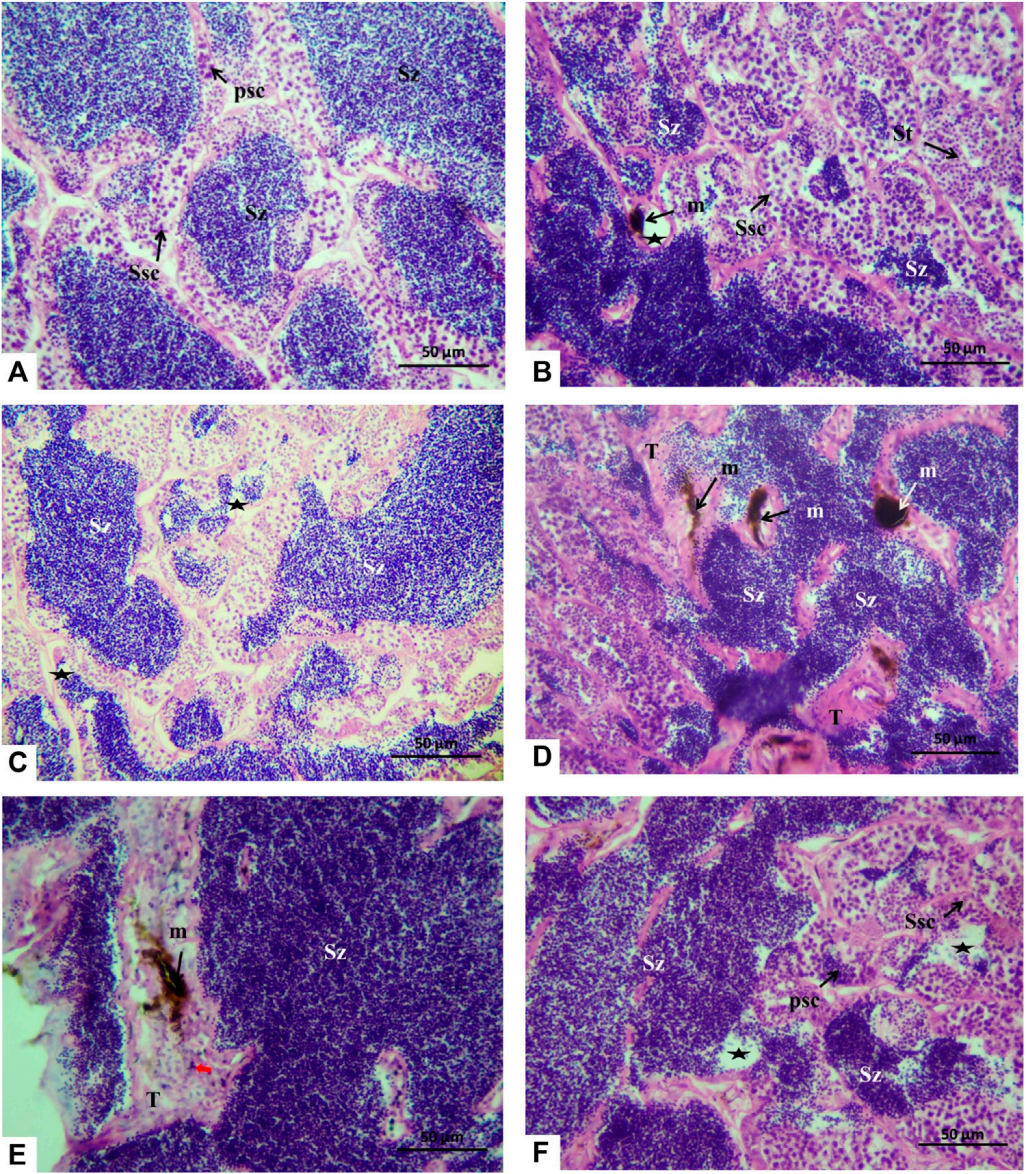
Histological cross section of Nile tilapia testes after 15 days of exposure. **(A)** Control group displaying the cystic organization with different spermatogenic cells, primary spermatocytes (Psc), secondary spermatocytes (Ssc), spermatids (St), and spermatozoa (SZ) in the lumen of the cysts. **(B)** H1 fish group showing few spermatozoa in the lumen of the testicular lobules (asterisks) and melanomacrophage center (m). **(C)** In the H1Lyc fish group, the sections displayed a slight deficiency of spermatozoa (asterisks). **(D, E)** H2 fish group, with different alterations, proliferation of interstitial tissue (T), and increasing melanomacrophage centers (m) and necrosis (red arrow), also exists. **(F)** H2Lyc fish group with few lacking spermatozoa (asterisks) (H&E staining).

The histology microphotographs of the testicular tissue displayed the control group with the cystic arrangement having different spermatogenic cell primary spermatocytes, secondary spermatocytes, spermatids, and spermatozoa (Figure 6A). For the fish group exposed to 10 μm/L Harness^®^, the testicular tissue showed a decrease in spermatozoa amount with few melanomacrophages (Figure 6B). While feeding a lycopene-supplemented diet (H1Lyc group), the sections were similar to those in control but displayed a slight insufficiency of spermatozoa in the lumen of the testicular lobules (Figure 6C). The higher-dose exposure of 100 μmol/L Harness^®^ induced different alterations, including the proliferation of interstitial tissue, increase in the melanomacrophage centers, and some necrosis (Figures 6D, E); however, lycopene supplementation (H2Lyc group) testis showed deficiency of spermatozoa from the lumen of the testicular lobules (Figure 6F).

## 4 Discussion

In the present study, the oxidative stress, thyroid toxicity, and reproductive toxicity of Harness^®^ to tilapia were studied. Oxidative stress is recognized as excess creation or deficient elimination of highly reactive molecules as ROS and reactive nitrogen species (Yonar, 2012). Oxidative injury is correlated to the failure of the antioxidant defense system for the exclusion of ROS inducing destruction of biomolecules like lipids, proteins, or DNA (Yonar et al., 2014).

The major antioxidant SOD and CAT defense enzymes are inducible enzymes. They can be prompted by an oxidative stress and represent the first line of resistance contrary to excessive free radicals in the body (Lin et al., 2009; Slaninova et al., 2009).

The antioxidant enzymes (CAT and SOD) inhibit oxidative stress, and the actions of these enzymes are commonly used to observe the risk of pesticides and herbicides (Jin et al., 2010). The results of the current study show that the SOD and CAT activities were altered throughout the experiment period. These changes point to the presence of oxidative stress, and the higher dose of 100 μmL Harness^®^/L induced significant elevation in both SOD and CAT, which might indicate increasing participation in free radicals’ exclusion. Similarly, in zebrafish, exposure to 10 and 100 μg/L acetochlor for 7 and 21 days induced plasma CAT, GPx, and SOD activities as an oxidative stress response activation (Zhang et al., 2020), while in zebrafish embryos, whole body CAT and SOD activities were stimulated as a response to exposure to acetochlor and/or the binary mixtures of bifenthrin and acetochlor for 14 days of treatment (Guo et al., 2020).

The present study declared that the TAC activity declined in fish groups exposed to the Harness^®^ (H1 and H2), while feeding a lycopene-supplemented diet restored the control levels. Similarly, a lycopene-supplemented diet can significantly upsurge the activity of TAC in Goldfish (*Carassius auratus*) (Meng et al., 2011).

In the current study, during Harness^®^ exposure, simultaneous feeding with lycopene-supplemented diets neutralized the TAC, SOD, and CAT enzyme activities and restored their control levels. Lycopene is regarded as a chemoprotectant agent due to its significant antioxidant scavenging action. In addition, it is also considered the most effective carotenoid used against biological ROS (Sahin et al., 2014). Abd El-Gawad et al. (2019) conveyed substantial augmentation of the antioxidant state of yellow perch fed a lycopene-incorporated diet for 60 days. They described a significant drop of the hepatic SOD and CAT activities. Several studies have suggested the use of lycopene as a strong antioxidant to attenuate the oxidative stress reactions in fish species subjected to insecticides (Dawood et al., 2020). Lycopene displayed an ameliorative effect against trichlorfon oxidative stress in common carp (*Cyprinus carpi*) (Yonar et al., 2020).

In the present study, only the higher dose of 100 μmol Harness^®^/L significantly increased serum T3, while the two exposure doses of 10 and 100 μmol Harness^®^/L induced elevation in the serum T4. Previous studies also verified that acetochlor is a thyroid-disrupting chemical (Helbing et al., 2006; Li et al., 2009; Jiang et al., 2015). Similarly, Yang et al. (2022) conveyed an increase in T4 and decline in T3 whole body levels for zebrafish larvae after acetochlor exposure. Acetochlor induced whole body T3 but not the whole body T4 in zebrafish larvae (Guo et al., 2020). The exposure of adult rare minnow to acetochlor for 21 days resulted in the suppression of blood T3 and T4 levels in male and female fish (Li et al., 2009). Acetochlor could disturb not only the secretion of thyroid hormones but also the expression of genes correlated to thyroid hormones (Li et al., 2009). In addition, acetochlor was found to elicit different responses in the secretion of THs, TH-linked crucial gene expression, and binding affinity to TRs that resulted in thyroid disruption, thus affecting the growth of zebrafish larvae (Xu et al., 2019).

The present results displayed histological alterations induced by Harness^®^ exposure. These alterations were more significant with the higher dose of Harness^®^ (100 μmol/L), while lycopene supplementation diminishes the thyroid cellular modifications prompted by Harness^®^. The enlarged thyrocyte cells of the Harness-exposed fish corroborated the increased level of serum T4 and T3 over the control levels. The size and height of the thyrocyte follicular epithelium are well thought as a marker of the thyroid secretory activity (Schnitzler et al., 2016; Ortiz-Delgado et al., 2019). These changes in both T3 and T4 levels and thyroid follicle histology have typically been used as direct endpoints to evaluate thyroid disruption in former studies on pesticides (Ortiz-Delgado et al., 2019). Our findings suggest that exposure to Harness^®^ could alter the HPT axis through oxidative stress initiation.

Lycopene supplementation reduced the thyroid cellular changes. Moreover, lycopene reduced the serum T3 and T4 levels induced by Harness^®^. These results suggest the effectiveness of lycopene in improvement of thyroid disorders through its antioxidant attributes. In this context, Abdul-Hamid and Salah (2013) reported that lycopene supplementation displayed an efficacy in amelioration of thyroid gland configurations against the insecticide deltamethrin, which may result from its antioxidant properties for albino rats. In addition, lycopene showed verified effectiveness in reinstating the thyroid structure and function after Aroclor 1254 exposure due to its antioxidant property (Ibrahim et al., 2021). In addition, it is strongly proposed that oral lycopene supplementation plays a significant role in diminishing the oxidative stress, where it is well thought as one of the best functional plant source antioxidants and widely used for defense counter to oxidative stress arbitrating cell and tissue injuries (Ibrahim et al., 2021).

In the current study, the reproductive impairments induced by Harness^®^ exposure for both male and female tilapia were studied. The results visibly revealed that Harness^®^ could affect tilapia ovarian and testicular progress. The two exposure concentrations of Harness^®^ caused oocyte atresia and ovarian growth obstruction, while they also caused spermatozoa deficiency and MMC in the testicular tissue. Consistently, individual exposure of acetochlor and its interactions with other pesticides affected the development of zebrafish gonads (Yang et al., 2022). In addition, an increase in the number of atretic follicles was detected in zebrafish ovaries subjected to acetochlor (Zhang et al., 2020). The exposure to 100 μmol/L acetochlor considerably repressed the ovarian growth in which the number of developed-stage oocytes significantly declined (Zhang et al., 2020).

The steroids T and E2 played a crucial role in the fish reproductive physiology regulation, which are the chief androgens and estrogens in vertebrates, and their key roles are to continue the normal growth of gonads (Lubzens et al., 2010), so their altered levels can affect the whole reproductive activity. Current results reported that the serum T levels exhibited significant (*p* < 0.05) reduction for exposed male and female fish, which increases with an increase in the concentration of Harness^®^, while for serum E2, a significant (*p* < 0.05) increase in E2 levels was observed in male fish and a decline in its level in female fish after herbicide exposure. Our results are in agreement with the published literature, which elucidated that the contents of E2 and T in male or female fish will change after pollutant exposure (Yang et al., 2022). In zebrafish, the exposure to acetochlor induced a significant decline in T testicular content and stimulated a significant increase in ovarian T content (Yang et al., 2022). Moreover, a decline in E2 ovarian content in zebrafish was reported after exposure to acetochlor (Yang et al., 2022). In contrast to these results, the low dose of acetochlor (1 μg/L) had an estrogenic influence and prompted E2 in zebrafish (Zhang et al., 2020). Similar effects on the T and E2 levels were observed in male and female walking catfish (*Clarias batrachus*) exposed to herbicide pretilachlor (Soni and Verma, 2020). Our findings of gonadal histology and sex steroids recommended that exposure to Harness^®^ reduced the capacity of gonadal resistance to oxidative stress and induced impaired gonadal growth.

In the present study, the lycopene-supplemented diet has a potential role in the alleviation of the reproductive damage induced by Harness^®^ exposure. It showed the ability to attenuate the histological impairments of gonads and, to some extent, restore the control levels of T and E2. In this context, lycopene is defined as one of the utmost encouraging antioxidants contrary to reproductive toxicity (Zhao et al., 2020). Consistent with our results, lycopene amended the induced reproductive dysfunction by enhancing T and E2 levels, sperm features, and histological characteristics in African catfish (*Clarias gariepinus*) (Sayed E.-D. H. et al., 2022). Lycopene supplementation efficiency has also been demonstrated in humans and animals with favorable results of improvement in male infertility and an increase in sperm count and viability (Durairajanayagam et al., 2014). Lycopene might relieve the seminiferous tubule and spermatogenic cell injuries in mice (Zhao et al., 2020) and defend against sperm and testicular injury in rats (Tripathy et al., 2017). Lycopene also enhanced sperm motility, number, density, and testosterone levels in mice exposed to pollutants (Boeira et al., 2015). Lycopene also displayed an ability to ameliorate the ovarian histological disorders in rats (Haq et al., 2022).

In light of the present findings, it can be concluded that herbicide Harness^®^ acts as an endocrine disruptor in *O. niloticus*; it alters the thyroid, gonadal tissues and T3, T4, and reproductive steroid hormonal hemostasis through the stimulation of oxidative stress. The present study also indicated that lycopene supplementation worked as a potent antioxidant and was able to alleviate oxidative stress and thyroid and reproductive toxicity caused by herbicide Harness^®^ exposure. The use of Harness^®^ in agriculture fields is a risk factor for the health and productivity of tilapia species as well as the health of human consumers; consequently, the usage of this herbicide in weed management demands to be considered cautiously.

## Data Availability

The data that support the findings of this study are available upon reasonable request.
